# Method for Bioimpedance Assessment of Superficial Head Tissue Microcirculation

**DOI:** 10.3390/s25237190

**Published:** 2025-11-25

**Authors:** Andrey Briko, Pavel Ryazantsev, Artem Gubko, Vladislava Kapravchuk, Sergey Shchukin, Boris Akselrod

**Affiliations:** 1 Biomedical Department, Bauman Moscow State Technical University, Moscow 105005, Russia; rpg18l188@student.bmstu.ru (P.R.); kapravchuk@bmstu.ru (V.K.); schookin@bmstu.ru (S.S.); 2 Petrovsky National Research Center of Surgery, Moscow 119435, Russia; gubko@artvig.ru (A.G.); aksel@mail.ru (B.A.)

**Keywords:** electrical impedance analysis, bioimpedance, microcirculation, intraoperative monitoring, tissue perfusion, cardiac surgery, non-invasive monitoring, laser doppler flowmetry

## Abstract

Assessment of microcirculation status during surgical interventions is of significant interest for monitoring tissue perfusion and controlling the effectiveness of systemic hemodynamics. This study investigated the applicability of the electrical impedance method for the quantitative assessment of changes in the blood supply to superficial head tissues during cardiac surgeries. Impedance signal recording was performed synchronously with laser Doppler flowmetry, allowing for the comparison of parameter dynamics reflecting microcirculatory processes. Analysis of a set of impedance parameters reflecting the amplitude and temporal characteristics of the pulse signal revealed consistent changes with microvascular indicators obtained by laser Doppler flowmetry. The most pronounced changes in impedance parameters were observed during transitions between key physiological states—induction of anesthesia, initiation of cardiopulmonary bypass, and its termination. This indicates the informativeness of the electrical impedance method for assessing the dynamics of scalp perfusion. The obtained results demonstrate its potential for non-invasive, continuous, and safe monitoring of microcirculation in superficial tissues in the operating room. This approach can be considered as an additional tool for comprehensive assessment of microcirculatory changes and improving the accuracy of tissue perfusion monitoring during cardiac surgeries.

## 1. Introduction

The microcirculatory unit is a system of vessels less than 100 μm in diameter, including capillaries, arterioles, and venules. This part of the vascular system plays a crucial role, as it is precisely where the tissues are supplied with oxygen and metabolic substrates necessary for cell functioning [1].

The goal of circulatory monitoring is to assess hemodynamic parameters and, if necessary, correct them. Modern trends in hemodynamic monitoring are aimed at reducing invasiveness and operator dependence, as well as implementing a staged approach that considers the phasic progression of critical conditions. This hemodynamic assessment is based on a comprehensive analysis of parameters, including cardiac output and myocardial contractility, venous return and preload, prediction of the response to fluid challenge, and afterload. However, the microcirculatory response and the adequacy of tissue perfusion under metabolic demands are rarely assessed.

However, the metabolic response of tissues and the reaction of microcirculation are crucial for understanding the pathophysiology of many conditions. Often, even with normal blood pressure and other central hemodynamic parameters, impairments in microcirculation can be observed. A number of studies have revealed a correlation with left ventricular ejection fraction, markers of myocardial injury, and neurohumoral factors [2]. However, a direct correlation with other central hemodynamic parameters (arterial pressure, heart rate, central venous pressure, etc.) is less pronounced. For instance, in cases of diffuse purulent peritonitis, microcirculatory disorders can be observed even with normal systemic hemodynamic indicators [3].

The kidneys and brain are the primary target organs affected by microcirculatory disturbances. This is due to their characteristically high blood flow per gram of tissue, complex capillary network (in the case of renal blood flow, the glomeruli and peritubular capillaries), sensitivity to changes in perfusion pressure and endothelial damage, dependence on blood flow autoregulation, and susceptibility to ischemia (Table 1) [4].

The most pronounced pathology of microcirculation is coronary microvascular dysfunction, which affects the small vessels of the myocardium and causes ischemia even in the presence of intact large coronary arteries. A growing body of evidence suggests that this process is systemic in nature and can be associated with cerebrovascular disorders, chronic kidney disease, retinopathies, liver diseases, pulmonary hypertension, and other conditions [5,6,7,8]. The relationship between cardiac and intestinal microcirculatory dysfunction is considered a key element of the systemic inflammatory response: damage to the intestinal barrier and microthrombosis intensify inflammation and exacerbate ischemic processes [9,10].

During cardiac surgeries and in critical conditions (e.g., sepsis), impairments in microcirculation, endothelial function, and tissue perfusion can develop even while systemic hemodynamic parameters remain normal. This emphasizes the necessity for direct and quantitative assessment of microcirculation, as it is the level that most accurately reflects the adequacy of oxygen and metabolite delivery to cells.

Intraoperatively, microcirculation can be significantly altered by perfusion pressure, circulating blood volume, anesthesia, and surgical manipulation. These fluctuations are often accompanied by reduced microvascular blood flow, vasodilation, or vasospasm. Therefore, monitoring microcirculation is as crucial as monitoring central hemodynamics.

Persistent impairment of microcirculation indicates a risk of tissue hypoxia and adverse outcomes. Early diagnosis of such changes allows for timely adjustment of fluid therapy and vasoactive drug dosages, which helps reduce postoperative complications and accelerate patient recovery [11]. In this context, microcirculatory monitoring is considered as a promising tool for personalized hemodynamic management, complementing traditional methods and providing a more thorough understanding of tissue perfusion dynamics [5,12].

A wide range of methods is currently used to assess the state of the microcirculatory bed. These methods must be non-invasive, safe for the patient, resistant to artifacts from the operating room environment, and suitable for continuous monitoring. The most common include biomicroscopy, near-infrared spectroscopy (NIRS), laser Doppler flowmetry (LDF), thermometry, and electrical impedance measurements (Table 2). Each approach is based on different physical principles and allows for the assessment of specific aspects of microcirculation—from visualizing microvessels and recording red blood cell movement to analyzing tissue oxygen saturation and changes in their electrical properties.

Biomicroscopy provides direct visualization of capillaries and red blood cell movement but is limited by a small observation area and high dependence on the quality of the optical system. NIRS allows for the determination of the relative content of oxy- and deoxyhemoglobin but characterizes microcirculation only indirectly, through tissue oxygenation parameters. Laser Doppler flowmetry remains the most widely used method for the functional assessment of microvascular blood flow; however, the measurement results are expressed in arbitrary units and require individual calibration. Thermometry, based on the detection of infrared radiation, is convenient for dynamic monitoring but has low specificity and is highly dependent on external conditions.

All the listed methods primarily assess the state of microcirculation based on the number of functional vessels and the velocity of red blood cell movement within them. However, organ functioning is largely determined by microcirculatory perfusion, that is, the effectiveness of volumetric blood supply to the tissues, which meets the metabolic demands of the cells. Quantitative assessment of this perfusion requires new approaches capable of reflecting not only the structural but also the functional characteristics of the microvascular bed.

Unlike traditional optical methods, the electrical impedance approach is based on measuring the complex electrical resistance of tissues, which depends on their blood filling and structural properties [20]. Methods for electrical impedance assessment of microcirculation record changes in tissue conductivity when a weak alternating current, applied to the skin surface, passes through them [21]. Analysis of the baseline and pulsatile components of impedance allows for the quantitative assessment of microcirculatory bed perfusion in absolute units (mL/min per 100 g of tissue) and the determination of vascular tone parameters. In combination with other rheogram characteristics, such as the rheographic index, this method can provide additional information about the condition of micro-vessel walls and the level of tissue perfusion [22,23].

The key advantage of the electrical impedance method is its non-invasive nature and applicability in real-time. An additional benefit is its electrical basis, which facilitates the seamless integration of impedance signal recording with other electrical monitoring techniques, particularly electrocardiography, which is routinely performed during surgeries. This integration establishes a foundation for the synchronous analysis of cardiac activity and tissue perfusion, thereby enhancing the informativeness of intraoperative monitoring and potentially enabling more precise interpretation of hemodynamic changes [24].

Despite its relatively low spatial resolution compared to direct imaging methods, electrical impedance analysis provides averaged information about tissue blood filling, which is valuable for the integral assessment of perfusion. Studies show that in vivo, impedance indices correlate with invasive parameters, such as the index of microvascular resistance, and can serve as functional markers of microcirculatory perfusion. Thus, the authors [10] demonstrated a significant correlation between electrical impedance and microvascular occlusion, confirming the diagnostic significance of the method in various pathophysiological conditions. 

Therefore, electrical impedance analysis represents a promising tool for continuous, quantitative, and objective monitoring of microcirculation. However, its clinical implementation requires experimental verification in real operating room conditions, comparison with established microcirculation assessment methods, and the identification of the most informative signal parameters.

The aim of this study was to evaluate the adequacy and reproducibility of the electrical impedance method for the quantitative assessment of microcirculatory perfusion in superficial head tissues in operating room conditions. To confirm the reliability of the measurement results, a comparison with laser Doppler flowmetry (LDF) data was conducted, allowing for the establishment of correlational dependencies between the parameters of the two methods. Additionally, informative impedance signal metrics were identified and clustered according to the stages of the surgical intervention, aiming to distinguish characteristic types of microcirculatory reactions.

## 2. Materials and Methods

### 2.1. Recorded Biosignals

During the study, synchronous recording of several physiological signals reflecting both local and systemic hemodynamic processes was performed. The recording was conducted simultaneously via two channels—frontal and peripheral—with temporal synchronization based on the electrocardiogram (ECG). This configuration enabled concurrent observation of microcirculation in superficial head tissues and systemic circulatory changes, allowing for cross-analysis of parameters at different regulation levels.

The primary measured signal was tissue impedance. Each channel recorded the full impedance signal Z(t), which was hardware-separated into two components: a baseline (Z_base_) and a pulsatile component. Baseline impedance represented a slowly varying component reflecting the overall level of blood filling in the examined area and its structural properties. Pulsatile impedance characterized rapid fluctuations synchronous with the cardiac cycle and reflected the dynamics of microcirculatory blood flow. Thus, the baseline component allowed for the assessment of stable changes in perfusion, while the pulsatile component captured instantaneous vascular reactions associated with the phases of cardiac activity.

An ECG was additionally recorded from the peripheral channel and used for the phase-locking of impedance signals to cardiac cycles. Combining the ECG with bioimpedance data ensured precise temporal synchronization of the signals and eliminated artifacts related to respiratory movements or electromagnetic interference.

To verify the impedance parameters and correlate them with established microcirculatory indicators, an LDF signal was recorded in parallel. The LDF data obtained were used to assess the correlation with impedance parameters and check the consistency of microcirculatory changes captured by the two independent methods.

### 2.2. Biosensor System and Measurement Setup

**Figure 1:**
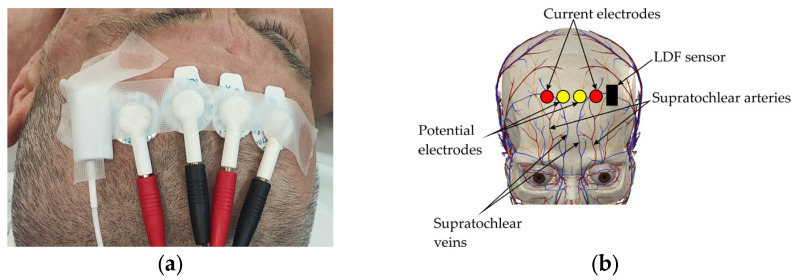
Electrode placement on forehead surface: (**a**) Electrode placement on subject’s forehead; (**b**) Projection of electrodes onto area of cranial vascular territory.

The choice of recording sites was based on anatomical and clinical considerations, as well as the need for minimal interference with the surgical procedure. The electrodes for the frontal bioimpedance channel were placed on the upper part of the forehead, closer to the hairline (Figure 1a). This positioning helped to minimize the influence of major facial vessels—the supratrochlear and supraorbital arteries—and increased the method’s sensitivity to changes in the microcirculatory perfusion of the superficial scalp tissues (Figure 1b).

In close proximity to the frontal electrode placement area, an LDF probe was positioned. This ensured that blood flow was recorded within the same vascular territory, enabling a direct comparison of the results from the two methods.

Based on previously conducted research, it was determined that to enhance the method’s sensitivity to microcirculatory changes, the electrodes should be placed at the minimum possible distance from each other. This placement increases the relative contribution of superficial tissue blood filling to the total bioimpedance signal by up to 90% [25].

Medico Electrodes MSGST-27 (Medico Electrodes International Ltd., Noida, India) disposable self-adhesive electrodes were used for recording. They were arranged in a Wenner tetra-polar configuration with an inter-electrode distance of 25 mm. Their use ensured stable contact quality, eliminated mechanical pressure on the tissues that can occur with reusable electrodes, and prevented displacement during surgery. Furthermore, the use of disposable electrodes promotes standardization of the methodology and, given positive results, simplifies its integration into clinical practice, as it does not require the development of specialized or expensive components.

**Figure 2:**
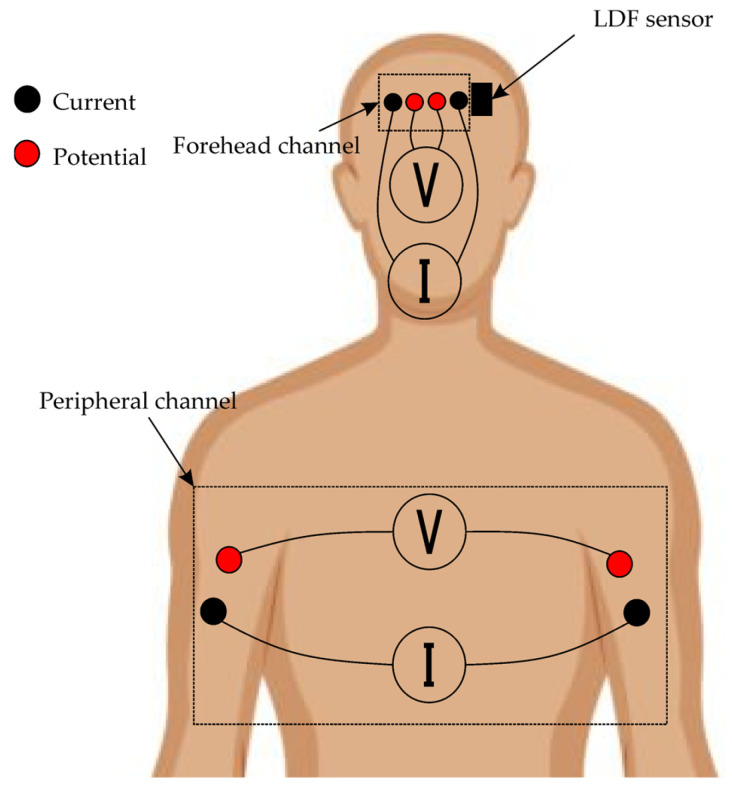
Layout of biosensor systems on patient’s body.

The peripheral channel was formed using four electrodes placed symmetrically on both upper limbs—two on each arm, positioned distally for the current electrodes and proximally for the potential electrodes (Figure 2). For the research, tetra-polar electrode assemblies [26] were used, ensuring reliable contact and resistance to displacement during prolonged recording. These assemblies consisted of disposable ECG electrodes with a contact diameter of 15 mm. This configuration ensured that the probing current passed through the thoracic cavity, enabling the recording of integral parameters of central hemodynamics, including stroke volume variations, as well as tracking respiratory phases based on characteristic impedance fluctuations.

### 2.3. Equipment

**Figure 3:**
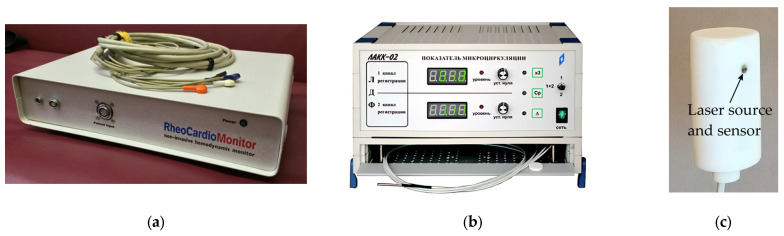
Images of equipment used: (**a**) Reocardiomonitor hardware system; (**b**) LAKK-02; (**c**) Appearance of LDF transceiver head.

The measurements were performed using the Reocardiomonitor hardware system (Research Institute of Biomedical Engineering, Bauman Moscow State Technical University, Moscow, Russia), shown in Figure 3a, and the LAKK-02 (Lazma Ltd., Moscow, Russia), shown in Figure 3b. The Reocardiomonitor device (Table 3) was used for recording bioimpedance signals and the electrocardiogram, while the LAKK-02 device (Table 4) was used for recording the laser Doppler flowmetry (LDF) signal. A specially designed transceiver head, conveniently placed on the patient’s body surface and developed by Lazma Ltd. (Moscow, Russia) for the purposes of this study (Figure 3c), was used as the LDF signal emitter and receiver.

To ensure temporal consistency between the recordings obtained from different devices, synchronization was performed based on the system time of the personal computer to which both devices were connected via separate interfaces. When recording began, timestamps were created, linked to the PC’s system time. This approach ensured the time referencing accuracy sufficient for analyzing the pulse and respiratory components of the signals.

### 2.4. Patients

The study involved 10 cardiac patients aged 41 to 74 years undergoing surgical treatment for valvular and aortic heart diseases. This patient category was selected because they frequently exhibit impairments in both systemic and microcirculatory hemodynamics, associated with altered myocardial contractility, fluctuations in perfusion pressure, and changes in vascular reactivity. This makes this group clinically significant for testing the sensitivity and informativeness of the bioimpedance method in assessing tissue perfusion.

Furthermore, cardiac surgeries are accompanied by significant intraoperative fluctuations in systemic circulatory parameters, creating natural conditions for studying microcirculatory responses and their relationship with macrohemodynamics. Thus, the selection of cardiac patients allowed for evaluation of the adequacy and reproducibility of bioimpedance indicators during dynamic changes in central hemodynamics.

Prior to the measurements, key anthropometric characteristics were recorded for each patient, including the thickness of the superficial soft tissue layer and head circumference, as these could influence the distribution of the electric field and the amplitude of the recorded impedance signal.

The soft tissue thickness in the electrode placement area was determined using ultrasonography (US) with a linear probe of a GE Vivid T9 (GE HealthCare, Chicago, IL, USA) ultrasound machine at a frequency of 7.5 MHz, measuring the distance from the skin surface to the aponeurosis. Head circumference was measured with a flexible measuring tape using a standard anthropometric technique—across the supraorbital ridges and the most prominent point of the occiput (Table 5).

### 2.5. Research Methodology

The study was conducted in the operating room during scheduled cardiac surgeries employing cardiopulmonary bypass (CPB). All patients were positioned supine, with any changes in body position during the procedure being minimal and dictated solely by surgical convenience.

Throughout the measurements, the stability of the electrical contact, skin surface temperature, and the absence of artifacts caused by surgical manipulations were monitored. Recording was temporarily suspended in situations where there was a risk of using electrosurgical instruments (electrocautery) or performing defibrillation, in order to prevent exposure to high-frequency interference and avoid damage to the measurement equipment. During these pauses, the connection to the electrodes remained intact, with only the connecting cable being unplugged from the device. This allowed for rapid reactivation of recording once the high-risk phase was completed.

To ensure the reproducibility of the results, the surgical procedure was divided into five clinically significant stages (Table 6). Signal recording was not performed during the CPB phase due to methodological constraints, as the absence of cardiac activity made it impossible to accurately identify cardiac cycles and calculate pulsatile parameters.

## 3. Data Processing

### 3.1. Software

Experimental data were collected using the standard software included with the Reocardiomonitor and LAKK-02 measurement systems, which enables the recording and saving of data in a digital format. The obtained time series of impedance, LDF, and ECG signals were saved as files for subsequent post-processing. The analysis was conducted using MATLAB R2022a and Python 3.11 (libraries: NumPy, Pandas, Matplotlib, Seaborn, Sklearn, SciPy) software suites.

The post-processing stage involved noise filtering and the extraction of informative pulsatile blood filling cycles using the Pan–Tompkins algorithm. Following this, data structures were created containing electrical impedance and LDF signals for the time segments identified in the previous step. Subsequently, characteristic metrics of amplitude, phase shifts, and correlations between the impedance and LDF signals were extracted for further statistical analysis.

### 3.2. Signal Metrics

**Figure 4:**
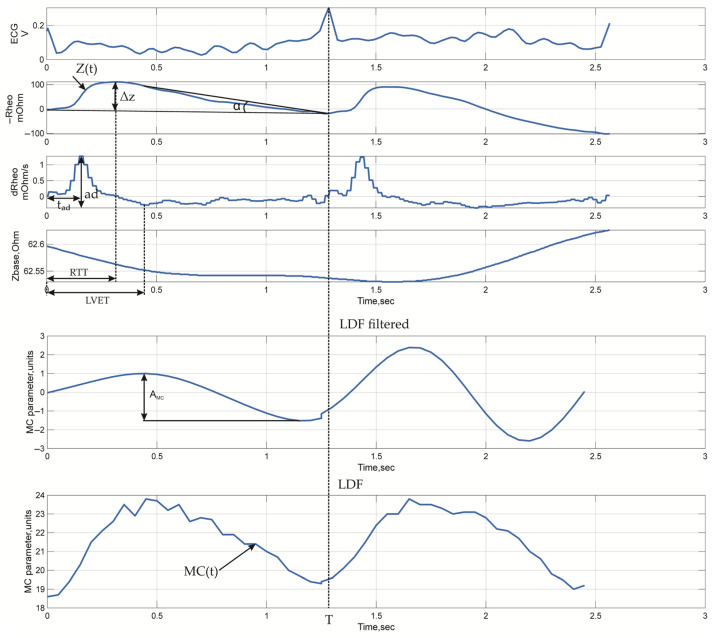
Typical time-domain waveforms with indicated measured parameters: ECG, electrical impedance signals recorded from arm surface (similar waveforms are observed for head channels), and LDF signals.

For the quantitative assessment of microcirculatory and systemic hemodynamic characteristics, sets of parameters were calculated to reflect the key features of the impedance signals (Figure 4). The calculated metrics included both basic indicators describing tissue blood filling and the dynamics of the pulsatile signal (Table 7), as well as more complex ones characterizing the synchronization of cardiac and peripheral processes (Table 7, the Complex parameters section). Parameters derived from the LDF signal, used for verification and comparison with impedance measurements, were identified separately (Table 7, LDF parameters section). Parameter calculation was performed for each cardiac cycle, the boundaries of which were determined by the R-R intervals of the electrocardiogram.

The selected parameters of the electrical impedance signals provide a comprehensive assessment of tissue perfusion processes and local hemodynamics. Basic indicators, including mean impedance and pulsatile amplitude, reflect the state of volumetric blood filling and depend on tissue hydration and blood inflow into the microcirculatory bed. Metrics characterizing the shape and dynamics of the impedance wave provide insight into the efficiency of arterial inflow, vascular wall tone and elasticity, while the temporal characteristics of the signal are related to the phases of the cardiac cycle and reflect the coordination of electrical and mechanical heart activity. For quantitative data interpretation, parameters characterizing microcirculatory perfusion were additionally calculated, including the rheographic index, which reflects vascular reactivity and relative changes in blood filling, as well as an integral indicator of the volumetric blood filling of the forehead tissues, based on modeling results (Appendix A). Its use allowed for the conversion of impedance data from relative to absolute physiological units (mL/100 g of tissue), ensuring comparability with traditional hemodynamic analysis methods.

Furthermore, complex indicators were calculated to assess the relationship between central and peripheral hemodynamics, as well as the spatial features of pulse wave propagation. The pulse wave transit time and the time it takes to propagate between recording zones characterize the speed of the pulse wave front and reflect the elasticity of the vascular wall and the tone of the microcirculatory vessels. The central–peripheral ratio index reflects the redistribution of blood flow between central and peripheral areas, serving as an indicator of systemic hemodynamic centralization. The ratio of baseline impedances is used to assess differences in tissue conductivity related to their hydration and blood filling and allows for tracking the dynamics of changes in the structural properties of tissues during the operation.

To verify the results of the impedance analysis, parameters calculated from the LDF signals reflecting the dynamics of microcirculatory blood flow were used. The primary indicator was the microcirculation index, which characterizes the average perfusion level in the measured tissue volume. Additionally, the amplitude of microcirculation index oscillations in the frequency range of 0.6–1.6 Hz corresponding to cardiac oscillations was assessed. According to data from study [27], this frequency range reflects the contribution of cardiac activity to the overall microcirculation spectrum associated with pulsatile blood flow fluctuations.

To analyze the interrelationships between various parameters and assess their informativeness, multidimensional clustering was performed. The calculated metrics were divided into four functional groups reflecting the spatial structure and levels of circulatory regulation:

(1) Impedance signal parameters recorded from the forehead surface;
(2) Impedance signal parameters recorded from the surface of the arms;
(3) Impedance signal parameters utilizing values from both recording channels;
(4) LDF signal parameters.

To normalize the surgical stage data, the OneHotEncoder function from the corresponding Python package was used. To scale the remaining numerical data, the StandardScaler function was applied. Clustering of the signal parameters was performed using the DBSCAN (Density-Based Spatial Clustering of Applications with Noise) algorithm. The characteristic parameters required for the algorithm’s operation were determined automatically by maximizing the silhouette coefficient as a metric of clustering quality. For visualization, dimensionality reduction was performed using the t-SNE (t-distributed Stochastic Neighbor Embedding) algorithm.

## 4. Results

**Figure 5:**
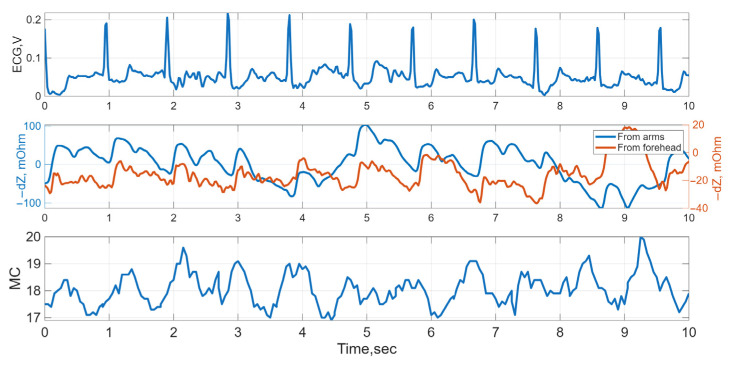
Typical time-domain waveforms: ECG, electrical impedance from arm and forehead surfaces, and LDF (MC, microcirculation) signals for Subject 2 at Stage 1.

Typical time-domain waveforms recorded during the study are shown in Figure 5.

Comparative results of volumetric blood filling, rheographic index, microcirculation index, and correlation coefficients between them, averaged across the data from 10 patients and by surgical stage, are presented in Table 8. For each parameter, median values and interquartile ranges (Me (Q1; Q3)), as well as sample averages, were calculated. Correlation coefficients were determined between the sets of values of the corresponding parameters calculated for the same surgical stage.

**Figure 6:**
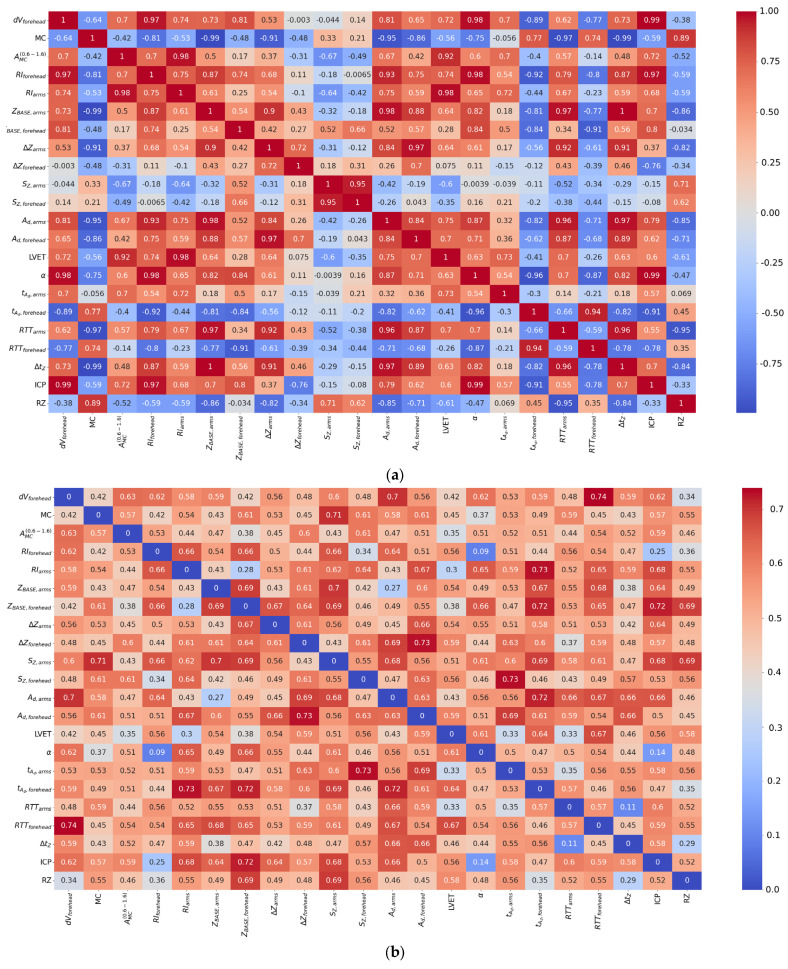
Heatmaps of correlation coefficients between parameters of electrical impedance and LDF signals: (**a**) mean values; (**b**) standard deviations, where values below 0.15 indicates stability of the dependency.

As seen in Table 8, strong negative linear correlation (Pearson correlation) was found between volumetric blood filling and the rheographic index and no such was found between bioimpedance and LDF signal parameters. After that, to assess the consistency of parameter dynamics across different surgical stages, pairwise correlation coefficients were additionally calculated between the stage-averaged values of the presented parameters and are shown in Figure 6a. A strong positive correlation was found between volumetric blood filling and the rheographic index (r = 0.97), confirming the consistency of impedance indicators reflecting tissue volumetric blood filling. Correlations between LDF signal amplitude and the rheographic index (r = 0.7) and between blood filling and LDF amplitude (r = 0.7) indicate a moderate coupling between local microcirculatory changes and systemic hemodynamic parameters.

Thus, despite the relatively weak correlation between the parameters of the impedance and LDF signals within individual stages (Table 8), when transitioning to stage-averaged values, an intensification of the interrelationships is observed, reflecting the coordinated dynamics of the systemic and microcirculatory components of circulation during the operation.

For a more detailed analysis of the relationships between the parameters of the electrical impedance and LDF signals, a full correlation matrix was constructed, presented as heatmaps (Figure 6). Unlike the data in Table 8, where correlational relationships were calculated within each surgical stage across the entire patient cohort, the heatmaps reflect the structure of interrelationships between all parameters obtained over the entire observation period, without additional averaging by stages. This approach allows for the assessment of global patterns of indicator interaction and the identification of general trends that persist despite individual patient differences.

Correlation coefficients were calculated using Pearson method, providing a quantitative assessment of the degree of linear relationship between the parameters. The heatmaps (Figure 6) show the mean values of the correlation coefficients between all calculated parameters and their standard deviations. The mean values reflect the degree of interconnectedness of the parameters in the aggregate data, while the map of standard deviations characterizes the variability of these relationships between patients, allowing for judgment on the stability of the identified correlational dependencies.

A comparison of the obtained heatmaps shows that when transitioning from the analysis within individual surgical stages to generalized relationships across the entire observation period, the key trends of consistency between systemic and microcirculatory indicators are preserved. Furthermore, the strength of the correlational relationships between the parameters of the electrical impedance and LDF signals increases with data aggregation, confirming the presence of coordinated dynamics of hemodynamic processes at both the systemic and local circulation levels.

From Figure 6, it follows that the specific blood filling showed a correlation with the LDF signal amplitude and with the rheographic index from the forehead. Furthermore, the majority of the electrical impedance signal parameters showed correlation with each other. 

**Figure 7:**
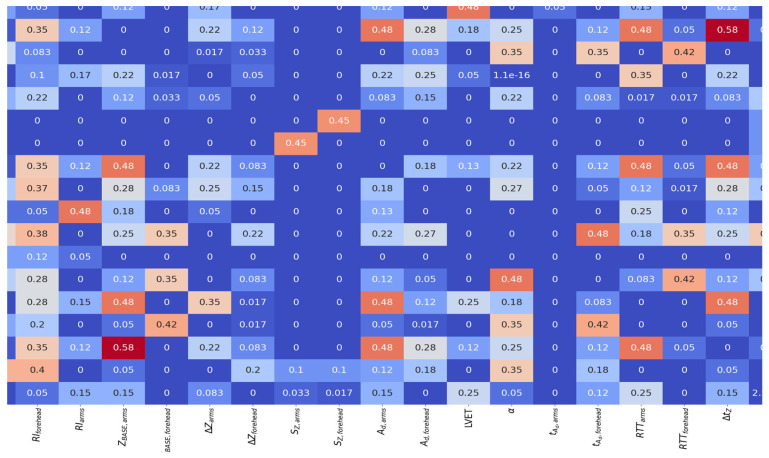
Heatmap of NMI (dimensionless) for electrical impedance and LDF signal parameters.

To determine the significance of both impedance and LDF signal parameters a mutual information (MI) calculation approach was used. This metric was introduced in [28] and could be used to determine whether parameters are independent or not. Then a matrix of normalized mutual information (NMI, which is MI that is normalized by the average entropy) was calculated. Consequently, a heatmap of NMI was constructed, presented in Figure 7.

From Figure 7, it follows that some of the electrical impedance signal parameters are unrelated to each other. In particular, it can be stated that the volumetric blood filling of the scalp tissues does not contain significant mutual information with any other signal parameter.

**Figure 8:**
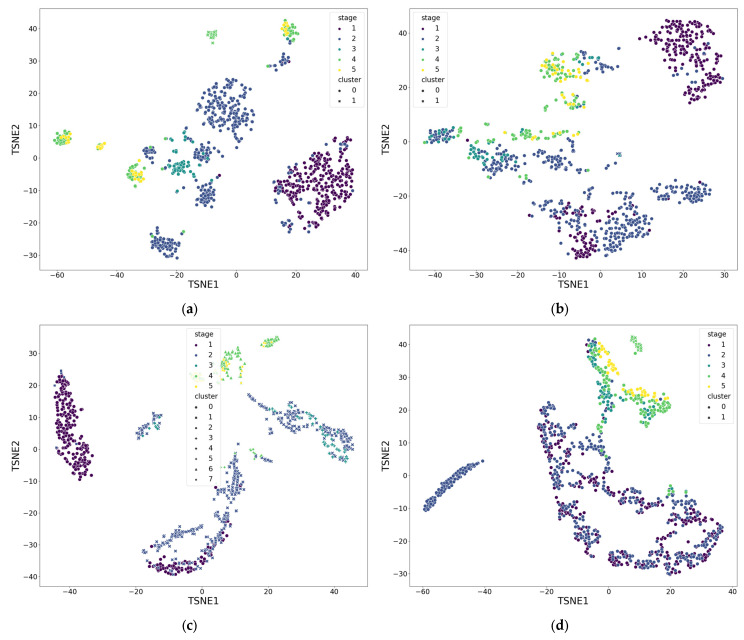
Result of parameter clustering by functional groups for patient 5: (**a**) Group 1; (**b**) Group 2; (**c**) Group 3; (**d**) Group 4. Stage—surgical stage, Cluster—label assigned by DBSCAN clustering algorithm. Groups are described in the point 3.2. Also a dimensionless reduction was used (variables TSNE1 and TSNE2).

The clustering results, averaged across all patients and presented in Table 9 and Figure 8 (where functional groups are described in the point 3.2), showed the presence of stable structural groups in all the studied datasets. For the impedance signals recorded from the forehead and arm surfaces, a pronounced data structuring was observed, indicating coordinated changes in hemodynamic characteristics within the different stages of the operation. This reflects the integral nature of the electrical impedance signal, which combines both systemic and regional components of circulation, including the microcirculatory contribution. A combined analysis of parameters from both recording channels revealed a more complex structure caused by the interaction of local and central mechanisms of perfusion regulation. The parameters of the LDF signal demonstrated greater distribution variability, which is consistent with their sensitivity to local microcirculatory fluctuations and individual characteristics of vascular reactions.

## 5. Discussion

The recorded electrical impedance signals exhibited substantial variations in amplitude and shape across different surgical stages, reflecting the action of different microcirculatory filling mechanisms. Indeed, transitions between phases (e.g., induction of anesthesia, initiation and termination of cardiopulmonary bypass) were accompanied by distinct changes in impedance parameters, indicating their sensitivity to perfusion shifts. The results of cluster analysis confirm this trend: impedance signal parameters grouped into a larger number of clusters (albeit with a slightly lower silhouette coefficient compared to LDF), evidencing the high sensitivity of the electrical impedance method to changes in both central hemodynamics and local microcirculation. This aligns with previous studies that demonstrated the high informativeness of impedance measurements for tracking blood flow dynamics in microvessels.

Despite some variability in absolute parameter values, significant correlations were found between the parameters reflecting microcirculation. Specifically, the correlation coefficient between the LDF signal amplitude and the calculated specific volumetric blood filling exceeded 0.9. This, along with a high correlation between the LDF amplitude and the rheographic index, confirms the validity of the proposed approach for the quantitative assessment of tissue perfusion. It is important to note that the calculated absolute values of volumetric blood filling (within ~1–8 mL/min per 100 g) correspond to literature data for normal skin perfusion. Thus, the electrical impedance method demonstrated an ability to quantitatively reflect changes in the microcirculatory bed, complementing existing optical techniques. Unlike the latter, which primarily assess blood flow velocity or the number of perfused capillaries, the impedance approach is directly related to the volumetric tissue blood filling—a key parameter determining the adequacy of oxygen and metabolite delivery. Our results indicate that a decrease in impedance corresponds to vasodilation, while an increase corresponds to vasoconstriction in the microvasculature, which is consistent with established physiological principles. This fact underscores the practical significance of the method: it can non-invasively track changes in vascular tone and perfusion in real-time, providing clinicians with new information about the patient’s microcirculatory status.

The observed relative divergence in the dynamics of the rheographic index and volumetric blood filling during certain transitions between stages warrants separate discussion. For instance, during the transition from the start of surgery to the phase of maximum hypnotic effect (induction of anesthesia with propofol), the rheographic index changed significantly (indicating vasodilation due to the anesthetic action), whereas the calculated volumetric blood filling demonstrated different dynamics. Consequently, the correlation between these two parameters decreased to ~0.72 over this time interval. This discrepancy may reflect the complex nature of blood flow redistribution: propofol causes systemic vasodilation, and the impedance signal might have captured both local and central effects. This phenomenon requires further investigation. It is necessary to further elucidate how such anesthesiologic interventions affect the relationship between impedance and microcirculatory parameters. Furthermore, correlating the identified impedance parameters with clinical outcomes—for example, with the presence or absence of postoperative complications in patients—is of significant interest. If a link can be established between microcirculatory disturbances (as per impedance monitoring data) and the development of complications, it would confirm the clinical significance and predictive value of the technique.

This study is of a pilot nature, with a limited sample size (10 patients) and a focus on cardiac surgeries. Although the obtained results demonstrate the fundamental feasibility and informativeness of the method, the statistical reliability of the conclusions could be strengthened by expanding the cohort. Future work plans to involve a larger number of patients to enhance statistical significance and account for inter-individual variability. Another direction for development is testing the methodology under controlled conditions using models of microcirculatory provocation. Experiments with local thermal and cold exposure to the forehead area using a Peltier element are planned. Such thermal and cold tests artificially induce vasodilation and vasoconstriction in skin vessels, respectively, which will allow for assessing the response of the impedance signal to control changes in perfusion. It is hypothesized that upon tissue cooling, impedance will increase due to reduced blood filling, while upon heating, it will decrease due to increased blood inflow, confirming the method’s ability to clearly capture changes in microcirculatory tone. Conducting these tests will serve as additional validation: the obtained data will help delineate the contribution of different regulatory mechanisms (myogenic, neurogenic, endothelial) to the formation of the impedance signal and optimize the frequency and algorithmic settings of the monitoring system. Finally, for full clinical validation of the methodology, testing in various patient groups with impaired microcirculation (e.g., in diabetes mellitus, septic conditions, peripheral vascular diseases, etc.), as well as comparison with the “gold standard” of perfusion assessment, is required. Such steps are planned as future work and will help define the limits of applicability for electrical impedance monitoring, facilitating the translation of the technology into clinical practice.

## 6. Conclusions

This study is the first to demonstrate the practical applicability of the electrical impedance method for the quantitative assessment of microcirculation in superficial head tissues during cardiac surgery. A mathematical model for calculating the volumetric blood filling of the microvascular bed from impedance data was developed and verified, confirmed on a sample of 10 patients undergoing cardiopulmonary bypass. A set of informative parameters for monitoring microcirculatory status was proposed, including complex characteristics of the impedance signal (specifically, the calculated specific volumetric tissue blood filling), as well as the amplitude and mean level of the LDF signal. It was shown that the dynamics of these parameters reflect perfusion changes at different stages of the operation, and their values are interrelated. When averaging indicators for each stage of the intervention, a strong correlation was found between volumetric blood filling, the rheographic index, and LDF amplitude, indicating consistency between impedance and optical assessments of microcirculation. At the same time, cluster analysis of the signals demonstrated higher sensitivity of impedance parameters to changes in physiological states and fluctuations in central and local hemodynamics compared to traditional LDF. Thus, the electrical impedance method is confirmed as a promising tool for non-invasive, continuous monitoring of microcirculation: it is capable of detecting even minor changes in tissue blood filling and vascular tone, complementing existing methods of perfusion control. The practical significance of the obtained results lies in the fact that impedance monitoring can be implemented to improve control over the state of the microcirculatory bed during operations and in the postoperative period, potentially reducing the risk of complications associated with impaired tissue perfusion.

Further development of this work will focus on expanding the clinical testing of the method and enhancing its diagnostic value. Plans include increasing the number of observations to improve the statistical power of the study, as well as conducting special functional tests (thermal and cold tests) to assess microcirculatory reactivity in healthy volunteers and patients. Furthermore, it is planned to correlate impedance parameters with clinical outcomes and conditions; for example, to compare parameter dynamics in patients with different complications or diagnoses affecting the microvascular bed. These steps will help to conclusively confirm the reliability and universality of the proposed methodology, as well as to determine its optimal areas of application in clinical practice.

## Figures and Tables

**Table 1:** Comparative analysis of organ perfusion parameters.

Organ	Relative Perfusion Parameters
Heart	0.08–0.10 mL/min/g
Brain	3.5 mL/100 g/min or 0.035 mL/min/g
Kidneys	4–5 mL/100 g/min or 0.04–0.05 mL/min/g
Intestines	1–1.5 mL/100 g/min or 0.01–0.015 mL/min/g

**Table 2:** Comparative characteristics of methods for assessing the state of the microcirculatory bed.

Method Name	Description	Signal Parameters	Advantages	Disadvantages
Biomicroscopy	A method for non-invasive diagnostics of blood vessels by obtaining images of the superficial layers of the region of interest containing microvessels [13,14]	Assessment of capillary geometry and red blood cell velocity in capillaries	Direct real-time visualization of the capillary state	Limited field of view, high technical requirements for the recording device, and consequently high equipment costs
Near-Infrared Spectroscopy (NIRS)	A method for determining oxyhemoglobin and deoxyhemoglobin contents based on the difference in their absorption capacities relative to the absorption capacity of soft tissues [15,16]	Concentration of oxy- and deoxyhemoglobin in the area under study is determined	Easy to implement, non-invasive	Measurement in relative units; influence of central hemodynamics due to greater penetration depth of radiation (compared to LDF)
Laser Doppler Flowmetry (LDF)	A method for measuring red blood cell velocity in the area under study [17,18]	Microcirculation index, proportional to the root mean square velocity of red blood cells in the area under study	Non-invasive, shallow penetration depth of radiation, ability to individually assess respiratory, myogenic, neurogenic, and endothelial frequency ranges of vascular tone regulation, no complex equipment required	Measurement in relative units, variability of results, lack of standardization, limited field of view, highly specialized equipment is required
Thermometry	Recording infrared radiation from the body surface	Temperature of the tissue area under examination	Ease of use, simultaneous signal acquisition from a large area, compatibility with other techniques, non-invasive, contactless	The method is more suitable for assessing changes, is highly dependent on environmental conditions, and requires highly specialized equipment
Electrical impedance	A method for assessing the hemodynamic characteristics of the vascular bed by measuring the complex impedance of tissues [19]	Baseline and pulsatile impedance	Capability to determine volumetric characteristics of blood filling, as well as parameters of vascular wall tone	The need to develop mathematical models for signal interpretation, dependence of measurement results on the quality of “electrode-skin” contact

**Table 3:** Technical specifications of reocardiomonitor.

Characteristic	Value
Number of impedance recording channels	2
Number of ECG channels	1
Channel sampling rate, Hz	500
Impedance measurement method	Tetrapolar
Probing current amplitude, mA	2.8
Probing current frequency, kHz	100
Baseline impedance measurement range, Ohm	1–240
Pulsatile impedance measurement range, mOhm	10–500
Input-referred electrical impedance noise value, mOhm	<0.5
Input-referred ECG noise value, μV	<5

**Table 4:** Technical characteristics of LAKK-02.

Characteristic	Value
Laser type	Single-mode semiconductor laser diode
Laser wavelength, nm	1064
Laser power at the fiber output, mW	<3.0
Doppler shift frequency recording bandwidth, Hz	20–24,000
Range of recorded red blood cell velocities, mm/s	0.3–6
Range of microcirculation index values, perf. units	0–99

**Table 5:** Patients’ anthropometric data.

Patient ID	Head Circumference, cm	Thickness of the Superficial Soft Tissue Layer, mm	Age, Years	Sex	Height, cm	Weight, kg
1	60	4	57	male	179	94
2	57	4	60	male	175	83
3	59	4	46	male	169	71
4	56	4	62	male	180	85
5	58	4	41	male	170	85
6	54	3	66	female	163	60
7	58	4	54	female	167	74
8	61	3	43	male	195	129
9	57	4	63	male	182	112
10	61	3	74	male	165	95

**Table 6:** Description of operation stages.

Stage Number	Description	Stage Duration Time Me (min; max) hh:mm:ss	Physiological Conditions
1	Patient’s condition before induction of general anesthesia	0:25:00 (0:10:00; 0:34:00)	Stable hemodynamics, spontaneous respiration
2	Maximum hypnotic effect	0:59:30 (0:43:00; 1:22:00)	Artificial lung ventilation, stabilization of systemic hemodynamics
3	Patient’s condition before initiation of cardiopulmonary bypass (CPB)	0:09:00 (0:02:00; 0:18:00)	Maintained anesthesia after thoracotomy, decreased body temperature
4	Patient’s condition after CPB	1:20:36 (0:51:00; 1:49:00)	Restoration of spontaneous circulation, normothermia
5	Patient’s condition after the end of surgery prior to transfer to intensive care unit	0:20:48 (0:16:30; 0:31:30)	Stable hemodynamics

**Table 7:** Parameters of electrical impedance signals.

№	Name	Formula	Calculation Group	Description
1	Baseline impedance		(1), (2)	Reflects non-cardiac tissue perfusion
2	Pulse impedance amplitude	$\Delta Z={Z\left(t\right)}_{max}-{Z\left(t\right)}_{min}$	(1), (2)	Reflects pulse wave tissue perfusion
3	Surface under pulse impedance curve	$S_{Z}=\int Z\left(t\right)dt$	(1), (2)	Is an indicator of stroke volume
4	Rheographic index	$RI=\Delta Z/Z_{BASE}$	(1), (2)	Reflects the volumetric blood flow
5	Differential rheogram amplitude	${A_{d}=\left(dZ/dt\right)}_{max}$	(1), (2)	Reflects the vessels’ activity
6	Left ventricular ejection time	LVET	(2)	Reflects cardiac activity
7	Pulse impedance curve fall angle	α	(1)	Reflects venous vessels’ activity
8	Time to peak of differential rheogram	$t_{A_{d}}$	(1), (2)	Reflects capillary activity
9	Volumetric blood filling (Appendix A)	dV	(3)	-
***Complex parameters***				
1	Pulse wave transit time for each channel	RTT	(1), (2)	Reflects vessels’ activity
2	Pulse wave transit time between channels	$\Delta t_{Z}={RTT}_{arm}-{RTT}_{forehead}$	(3)	Reflects the difference in macro- and microvessels’ activity
3	Central–peripheral ratio index	$ICP={\Delta Z}_{forehead}/{\Delta Z}_{arm}$	(3)	Reflects the difference in pulse wave blood perfusion
4	Baseline impedance ratio	$RZ=Z_{BASE}^{forehead}/Z_{BASE}^{arm}$	(3)	Reflects the difference in non-cardiac perfusion
***LDF parameters***				
1	Microcirculation index	MC	(4)	Is a value proportional to the multiplication of the amount of the blood cells and theirs root mean square velocity
2	Microcirculation index amplitude	$A_{MC}^{\left(0.6-1.6\right)}=\sqrt{\int_{0.6}^{1.6}S_{MC}\left(f\right)df}$	(4)	Reflects the changes in MC value due to the pulse wave blood perfusion

**Table 8:** Values of volumetric blood filling, rheographic index, LDF signal amplitude, and pairwise correlation coefficients, M ± Std, Me (Q1; Q3); *, *p* < 0.05 according to Mann–Whitney test compared to previous stage.

Stage Number According to Table 6	${dV}_{forehead}$, mL/min by 100 g. of the Tissue	${RI}_{forehead}$, *10^3^ Units	$A_{MC}^{\left(0.6-1.6\right)}$, perf. Units	$r_{{RI}_{forehead},{dV}_{forehead}}$	$r_{{RI}_{forehead},{A_{MC}^{\left(0.6-1.6\right)}}_{forehead}}$	$r_{{dV}_{forehead},A_{MC}^{\left(0.6-1.6\right)}}$
1	5.94 ± 7.28, 5.77 (3.30; 8.38)	0.86 ± 0.00, 0.88 (0.47; 1.18)	1.13 ± 0.91, 0.81 (0.54; 1.40)	−0.98 ± 0.02, −0.98 (−0.99; −0.97)	0.12 ± 0.16, 0.12 (0.01;0.16)	−0.12 ± 0.16, −0.11 (−0.17; 0.011)
2	7.35 ± 9.54, 5.83 (2.59; 11.89) *	0.89 ± 0.00, 0.80 (0.38; 1.40)	2.07 ± 2.08, 1.68 (0.87; 3.12) *	−0.97 ± 0.03, −0.98 (−0.99; −0.97)	0.03 ± 0.14, 0.03 (−0.09; 0.11)	−0.01 ± 0.15, −0.03 (−0.12; 0.11)
3	7.75 ± 10.89, 6.66 (2.85; 9.56)	0.75 ± 0.00, 0.84 (0.42; 1.23)	1.53 ± 1.05, 1.12 (0.74; 2.30) *	−0.98 ± 0.02, −0.98 (−0.99; −0.96)	0.05 ± 0.10, 0.04 (−0.01; 0.1)	−0.03 ± 0.11, −0.04 (−0.12; 0.04)
4	4.57 ± 21.77, 5.13 (0.21; 9.24) *	0.26 ± 0.00, 0.56 (−0.02; 1.06) *	1.29 ± 1.02, 1.07 (0.69; 1.65) *	−0.98 ± 0.02, −0.99 (−0.99; −0.97)	0.18 ± 0.15, 0.20 (0.07; 0.26)	−0.17 ± 0.16, −0.17 (−0.27; −0.07)
5	5.08 ± 12.92, 3.22 (1.71; 6.79) *	0.28 ± 0.00, 0.29 (−0.17; 0.58) *	0.88 ± 0.64, 0.75 (0.47; 1.09) *	−0.99 ± 0.03, −1 (−1; −1)	−0.07 ± 0.41, 0.06 (−0.12, 0.15)	0.07 ± 0.41, −0.06 (−0.12; 0.11)

**Table 9:** Clustering results of electrical impedance and LDF signal parameters.

Functional Groups	Range of Number of Clusters Me (min; max)	Silhouette Coefficient Values M, std (min; max)
1	2 (2; 3)	0.468, 0.137 (0.148; 0.586)
2	2 (2; 4)	0.517, 0.142 (0.222; 0.798)
3	4 (2; 13)	0.340, 0.164 (0.195; 0.678)
4	4 (2; 6)	0.452, 0.229 (0.149; 0.730)

**Figure A1:**
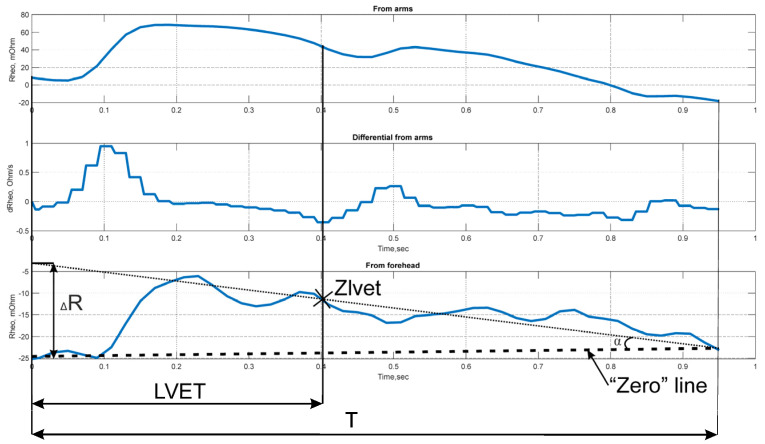
Determination of ∆R.

## Data Availability

The data obtained and analyzed during this study are available from the corresponding author upon reasonable request.

## Appendix A

### Appendix A.1. Formulas for Calculating Volumetric Blood Filling of Forehead Tissues

The relationships used to calculate the volumetric blood filling of the superficial forehead tissues incorporate the value of the specific electrical resistivity of the tissues (*ρ*_tissue_), which directly determines the value of the recorded impedance signal [29]. To evaluate the electrical properties of the superficial head tissues from experimental data, a direct search method was applied, based on comparing the measured impedance values with the results of finite element modeling. The goal of this approach was to determine the value of *ρ*_tissue_ that minimizes the mean squared error functional between the calculated and experimental data.

The calculations utilized individual anthropometric parameters of the volunteers, including the thickness of the superficial soft tissue layer and head circumference. This allowed for the consideration of the specific structural features and tissue conductivity of each individual patient. This approach ensured personalized modeling of the electric field distribution and enhanced the accuracy of recovering the specific tissue resistivity. This methodology is described and demonstrated in detail in the authors’ previous works [21].

It is worth noting that for the equipment used, the following holds true: at a probing current frequency of 100 kHz, the primary contribution to the impedance signal comes from the active resistive component R. Therefore, during model development, it was assumed that the recorded impedance Z is the active resistance of the tissue, R_tissue._

To calculate the volumetric blood filling, a model based on the representation of tissue and blood as parallel-connected resistors was used. The following computational relationships are valid for this model:

The tissue is represented as a “cube” with a mass of 100 g and arbitrary dimensions. Its resistance is calculated using Equation (A1):

(A1) $$R_{tissue}=\rho_{tissue}\frac{l^{2}}{V_{tissue}}$$

Based on the volume V of blood flowing into the tissue during one cardiac cycle, its resistance is calculated by Equation (A2):

(A2) $$R_{blood}=\rho_{blood}\frac{l^{2}}{V}$$

The total resistance of the “tissue + blood” system $R_{tissue+blood}$ is calculated as a parallel connection of resistors;

The change in resistance ∆R due to blood filling is calculated using Equation (A3):

(A3) $$∆R=R_{tissue+blood}-R_{tissue}$$

The change in the specific tissue resistivity is calculated using Equation (A4):

(A4) $$∆\rho =\frac{S_{tissue}\cdot ∆R}{l}$$

Based on Formulas (A1)–(A4), the specific volumetric blood filling of the superficial soft tissue layer is determined by expression (A5):

(A5) $$∆V\left(\frac{mL}{min}by\;100\;g.\;of\;the\;tissue\right)=\;\frac{0.1\cdot ∆\rho \cdot \rho_{blood}\cdot HR\cdot 10^{6}}{\rho_{tissue}\cdot \rho_{mech.tissue}\cdot \left(∆\rho -\rho_{tissue}\right)}$$

where HR denotes heart rate (beats/min), $\rho_{mech.tissue}$ denotes tissue density (for the superficial layer 975 kg/m^3^ [29]), and ∆ρ and $\rho_{tissue}$ are determined from the experimental pulsatile ∆R and baseline *R* impedance using the dependence obtained from numerical modeling.

An approach based on the analysis of the electrical impedance signal waveform over a single cardiac cycle (Figure A1) was used to determine ∆R.

Physiologically, the measured value of ΔR represents the volume of blood ejected into the microcirculatory bed of the examined tissue area.
